# Potential biomarkers and therapeutic targets in juvenile spondyloarthritis patients

**DOI:** 10.1186/1546-0096-12-S1-P44

**Published:** 2014-09-17

**Authors:** Lovro Lamot, Mandica Vidović, Marija Perica, Lana Tambić Bukovac, Kristina Gotovac, Fran Borovečki, Miroslav Harjaček

**Affiliations:** 1 University of Zagreb School of Medicine, Zagreb, Croatia; 2 Pediatric and Adolescent Rheumatology, Children's Hospital Srebrnjak, Zagreb, Croatia; 3 Department for the Functional Genomics, Center for Translational and Clinical Research, University of Zagreb School of Medicine, Zagreb, Croatia

## Introduction

To understand disease mechanism and close the gap between genotype and phenotype, majority of studies today use the high-throughput methods that allow us to study genes on a global scale. One of these methods is expression profiling which generates a “snapshot” of cellular activity at the time of analysis, telling us exactly what processes are occurring. By comparing disease and control samples it is possible to elucidate the processes contributing to disease and how they are altered. To date a number of disparate expression profiling studies in patients diagnosed with spondyloarthritis (SpA) have been undertaken, but results of these studies were often inconsistent. To the best of our knowledge, there has been no expression profiling study with RNA isolated from whole blood in a cohort of patients diagnosed with juvenile spondyloarthritis (jSpA) using ILAR criteria for enthesitis related arthritis (ErA), with known HLA genotype and calculated odds ratio (OR) for disease development. Further on, there was no study in which expression of selected genes was independently confirmed in new cohorts of untreated and treated patients diagnosed with jSpA, as well as with other forms of juvenile idiopathic arthritis (JIA).

## Objectives

The aim of the present study was to identify and confirm gene signatures and novel biomarkers in various cohorts of untreated and treated patients diagnosed with jSpA and other forms of JIA.

## Methods

Total RNA was isolated from whole blood of 45 children with known HLA genotype, calculated odds ratio for disease development and diagnosis of jSpA according to ILAR criteria, 11 children with oligo- and polyarticular forms of JIA, as well as 12 age and sex matched control participants without diagnosis of chronic inflammatory disease. DNA microarray gene expression with Affymetrix GeneChip was performed in 11 patients with jSpA and in four healthy controls, along with bioinformatics analysis of retrieved data (DAVID, GSEA, IPA). Carefully selected differentially expressed genes (TLR4, NLRP3, PTPRN2, CXCR4, DUSP6, TNFSF4, MAP2K2, MAPKBP1, MYST3, PTPN12) where analyzed by qRT-PCR in all study participants.

## Results

Microarray results and bioinformatics analysis revealed 745 differentially expressed genes involved in processes such are antigen recognition and activation of immunological response, migration of inflammatory cells and regulation of the immune system. qRT-PCR analysis of selected genes confirmed data universality and specificity of expression profiles in jSpA patients.

## Conclusion

The present study has identified differences in the expression of genes related to various processes responsible for jSpA development. Among these, the genes of highest importance, which showed consistent expression in study patients diagnosed with jSpA, were TLR-4 and NLRP3, which can lead to the development of systemic autoimmunity, as well as CXCR4 and PTPN12, which can drive other processes important for pathophysiology of jSpA. We strongly believe that our results represents an important step toward a better understanding of the molecular mechanisms and complex pathophysiology of jSpA, while highlighted genes and their products could have a prognostic value and potential therapeutic use.

## Disclosure of interest

None declared.

